# Barriers to completing colonoscopy after a positive fecal occult blood test

**DOI:** 10.1186/s13584-021-00444-2

**Published:** 2021-02-11

**Authors:** Revital Azulay, Liora Valinsky, Fabienne Hershkowitz, Einat Elran, Natan Lederman, Revital Kariv, Benjamin Braunstein, Anthony Heymann

**Affiliations:** 1 Meuhedet Health Care, 5 Pesach Lev, Lod, Israel; 2 Public Health Nursing, Ministry of Health, Jerusalem, Israel; 3 Maccabi Healthcare Services, Tel aviv, Israel; 4 Faculty of medicine University of Tel Aviv, Tel Aviv, Israel

**Keywords:** Cancer, Positive colorectal cancer screening, Colonoscopy, Adherence

## Abstract

**Background:**

Colorectal cancer leads to significant morbidity and mortality. Early detection and treatment are essential. Screening using fecal occult blood tests has increased significantly, but adherence to colonoscopy follow-up is suboptimal, increasing CRC mortality risk.

The aim of this study was to identify barriers to colonoscopy following a positive FOBT at the level of the patient, physician, organization and policymakers.

**Methods:**

This mixed methods study was conducted at two health care organizations in Israel. The study included retrospective analyses of 45,281 50–74 year-old members with positive fecal immunochemical tests from 2010 to 2014, and a survey of 772 patients with a positive test during 2015, with and without follow-up. The qualitative part of the study included focus groups with primary physicians and gastroenterologists and in-depth interviews with opinion leaders in healthcare.

**Results:**

Patient lack of comprehension regarding the test was the strongest predictor of non-adherence to follow-up. Older age, Arab ethnicity, and lower socio economic status significantly reduced adherence. We found no correlation with gender, marital status, patient activation, waiting time for appointments or distance from gastroenterology clinics. Primary care physicians underestimate non-adherence rates. They feel responsible for patient follow-up, but express lack of time and skills that will allow them to ensure adherence among their patients. Gastroenterologists do not consider fecal occult blood an effective tool for CRC detection, and believe that all patients should undergo colonoscopy. Opinion leaders in the healthcare field do not prioritize the issue of follow-up after a positive screening test for colorectal cancer, although they understand the importance.

**Conclusions:**

We identified important barriers that need to be addressed to improve the effectiveness of the screening program. Targeted interventions for populations at risk for non-adherence, specifically for those with low literacy levels, and better explanation of the need for follow-up as a routine need to be set in place. Lack of agreement between screening recommendations and gastroenterologist opinion, and lack of awareness among healthcare authority figures negatively impact the screening program need to be addressed at the organizational and national level.

**Trial registration:**

This study was approved by the IRB in both participating organizations (Meuhedet Health Care Institutional Review Board #02–2–5-15, Maccabi Healthcare Institutional Review Board BBI-0025-16). Participant consent was waived by both IRB’s.

## Background

Colorectal cancer (CRC) is an important contributor to morbidity and mortality worldwide. Early detection of cancer or precancerous polyps together with prompt, appropriate treatment is of major importance in cancer control [1]. Although population based screening for colorectal cancer is recommended by the European Union, there is room for improvement in program implementation in most European countries.

Recommended strategies for early detection for population screening are either fecal occult blood testing (FOBT) yearly or colonoscopy every 10 years from the age of 50 to 74 years [2]. National CRC screening programs are available in many countries (Canada, Britain, France, Italy, Australia, Israel, Sweden and Denmark), and participation rates vary between 30 and 60%, although specific interventions have led to participation rates of up to 80% [3–9].

Most screening programs are based on a fecal immunochemical test (FIT) for occult blood, with colonoscopy follow-up after a positive finding. However, adherence to follow-up is surprisingly low, resulting in sub-optimal screening programs, even when initial program participation is high. In studies conducted in the US, only 49% of participants with a positive FOBT result completed a follow-up colonoscopy at 3 months, and 59% at 1 year [10, 11]. In Ontario, Canada, 75% completed follow-up at 6 months, even though guidelines specify a follow-up period as 60 days [12, 13], and in Japan 60% completed follow-up at a national level [14]. Targeted interventions have been shown to increase follow-up up to 90% in small studies in France, Britain and Sweden [15–18]. In Israel although participation rates in the national CRC screening program are high (64%) the follow-up colonoscopy rate within the recommended 3 months are 40, and 70% at 1 year [8].

Several barriers to follow-up after positive screening tests have been described in the literature [19, 20]. These focus on four areas: information technology (IT) [21, 22], healthcare organizations [23, 24], physician behavior [25–28] and patient emotional and cognitive factors [29–34]. Socio-demographic variables have also been found to be associated with both CRC screening and follow-up including gender [14] and age [35].

Completion of all required steps in a CRC screening program is essential to maximize the potential reduction in morbidity and mortality associated with this cancer. A large proportion of participants in screening programs do not complete follow-up after a positive FOBT result [22]. The aim of this study is to identify the organizational, clinical, environmental and personal barriers to follow-up in this population in order to provide specific, patient oriented, systematic and evidence-based interventions to improve follow-up of positive fecal occult blood screening results.

## Methods

In Israel, National Health Insurance Law covers all citizens for a defined set of healthcare needs, provided by one of four Health Care Organizations (HCO). Members can select any of the four, and can select to extend their coverage through complementary insurance in the same HCO. In addition, citizens can also purchase private health insurance from a number of insurance companies. The HCO is responsible for all care, including preventive medicine and health promotion, and services are managed within HCO clinics. Services are provided by both directly employed physicians who see patients in HCO clinics, and contract physicians who generally see patients in their own practice. CRC screening is the responsibility of primary physicians, supported by clinic staff. FIT kits are usually provided to the patients by nurses, laboratory technicians or physicians, and all test results are sent to the patient’s primary physician.

This mixed methods study was conducted in two HCO’s in Israel (Meuhedet and Maccabi Healthcare Services) who provide care for 3.2 M members, 40% of the Israeli population. The study was conducted between 2015 and 2017, and included retrospective data analyses, a patient survey, physician focus groups and in depth interviews with policymakers. The study was approved by Institutional Review Boards in both organizations (Meuhedet #02–2–5-15, Maccabi BBI-0025-16) and was funded by a research grant from the Israeli Institute for Health Policy Research.

### Study design

1. Retrospective data analysis (2010–2014)

In Maccabi and Meuhedet. There are 580,000 members aged 50–74. Every year all of these members are invited to have a FIT, and every year, 60% have either had a FIT in the previous year or a colonoscopy in the previous 10 years. Once a patient has a positive FIT they are excluded from subsequent years, as once the result is positive, the appropriate test is a colonoscopy and not a repeat FIT. We compiled a dataset of all members aged 50–75 who had a FIT between the years 2010 and 2014 and had a positive result (approximately 6% of FITs for each year). We obtained clinical and demographic information from each HCO’s central database which includes all coded data from patient electronic medical records (EMR), including age, gender, home address, medical test and procedure information, and co-morbidities. Ethnic association was defined using the characteristics of the clinic where the participants were registered, as this is not available in the EMR. We used the participant’s home address to define four variables. 1. Socio-economic status (SES), using the Israeli Bureau of Statistics (IBS) SES levels [36] based on geographic areas; SES levels range from 1 to 20 with 20 being the highest SES. 2. Peripherality Index of Local Authorities - a variable used by the IBS that describes the distance between any locality and the closest financial activity center, and is used as an indicator of proximity to services - the lower the index the more central the location; 3. Access to colonoscopy as the distance between each home address and the closest gastroenterology clinic where colonoscopies are performed using a geographic information system (GIS) application (MapInfo Pro, V. 15.0, Pitney Bowes Software); 4. District: Both HCO’s provide services via regional directorates, dividing Israel into 4 districts- the northern region, the central region including Tel-Aviv, the eastern region surrounding Jerusalem, and the south of Israel. These regions differ both in access to medical services in general, and ethnic composition. We obtained colonoscopy availability by contacting all gastroenterology clinics (approximately 100) and documenting the next available appointment for colonoscopy in each one.

Using SPSS for Windows v 24.0 we conducted analyses of two dependent variables -adherence to colonoscopy within 12 months of a positive FIT (dichotomous) and the period between the positive FIT and colonoscopy (continuous). Chi-squared and t-tests were used to compare the groups with respect to categorical and continuous variables, respectively. Multivariate analyses were conducted using logistic regression for predicting adherence to colonoscopy, adjusted for variables found to be related to adherence at the univariate level. A 5% level of significance was used for all statistical tests.

2. Patient survey (Positive FIT patients during 2015)

During 2015, 2521 members aged 50–74 in both HCO’s had a positive FIT. In order to have a final sample of respondents that was sufficient for our analysis (at least 500), and assuming a 30% response rate, we selected 1522 using random numbers, for the survey. Exclusion criteria were: no personal or family history of CRC or high risk for CRC. We collected information using a telephone questionnaire that included demographic information, patient recall of information regarding the FIT, patient experience regarding the process (e.g. who provided the test, had they received any explanations regarding the test), and the Patient Activation Measure (PAM) short version [37].

The PAM was developed to evaluate the degree of patient activation among patients with chronic disease [38] and has since been shortened, and validated in Hebrew [39]. We translated and validated the PAM in Arabic, English and Russian, for the purpose of this study [40]. The questionnaire includes 13 items, and results are presented in four categories- from low patient activation (1) to high (4).

Demographic variables included education level, ethnicity (Jewish, non-Jewish), religiosity (the degree of adherence with religious practices) and country of birth (Israel or other countries), and health insurance status. The purchase of complementary or private insurance choices, as opposed to the basic healthcare coverage, are dependent on income as well as personal prioritization.

3. Focus groups and in-depth interviews (2016–2017)

The aim of the qualitative part of this study was to explore physicians’ views on the issue of follow-up after a positive FIT, whether this is a serious problem, and the perceived barriers to follow-up from different perspectives. In order to do this, we conducted three focus groups between September 2016 and January 2017. Two groups consisted of approximately 15 primary care physicians each, one group from each HCO in the study. One group consisted of 10 gastroenterologists, most of whom work with both organizations. The topics that were introduced in the group discussions were:

1. What promoting factors and what barriers influence follow-up after a positive FIT (access, availability, organizational culture, and proactivity)?
2. What are patient attitudes regarding follow-up, and how do these affect patient behavior, as perceived by physicians?

We also conducted eight in-depth interviews with senior policy-makers within the Israeli health care system, including public health experts, family practice and gastroenterology professional leaders.

All focus groups and interview content was recorded and subsequently analyzed using thematic analyses.

## Results

### Retrospective data analysis

The study population included 45,281 who had a FIT between the years 2010 and 2014 and had a positive result. Table 1 describes the study population variables, and the proportion of participants in each group who had a colonoscopy up to 1 year after a positive FIT.

**Table 1:** Study population, time to colonoscopy and adherence to colonoscopy at 1 year-Univariate analysis

Variable	Values	N	Population distribution (%)	Adhered to colonoscopy (12 months, %)	***p***-value for adherence	Mean number of days to colonoscopy (SD)	***p***-value for time
Gender	Male	24,082	53.2	63.3	0.170	77.7(69.8)	0.026
	Female	21,199	46.8	64.0		75.9(67.3)	
Age group	50–55	9626	21.3	65.1	0.001>	75.6 (68.2)	0.001>
	56–60	9063	20.0	64.1		75.0(66.9)	
	61–65	10,668	23.6	64.6		76.7(68.7)	
	66–70	8853	19.6	63.7		77.4(70.0)	
	71–75	7071	15.6	59.5		80.9(69.5)	
SES^a^	1–5	3540	8.1	54.2	0.001>	83.5(74.0)	0.001>
	6–10	17,530	40.0	64.1		77.4(69.2)	
	11–15	18,307	41.8	65.6		76.7(68.2)	
	16–20	4459	10.2	66.5		72.3(65.7)	
Ethnicity	Jewish Orthodox	5946	16.4	61.7	0.001>	76.6(69.4)	0.007
	Arab	2112	5.8	44.8		84.0 (79.0)	
	Jewish Secular	28,271	77.8	63.6		77.0(68.0)	
District^b^	Central	12,456	23.0	61.4	0.001>	78.3(65.8)	0.069
	Southern	17,054	43.5	67.7		77.4 (69.9)	
	Northern	9881	20.1	60.9		77.4(68.9)	
	Jerusalem	5126	13.4	60.0		74.1 (68.8)	
Year of FIT	2010	6904	15.2	55.8	0.001>	88.9(73.9)	0.001>
	2011	8551	18.9	61.8		83.6(72.2)	
	2012	9185	20.3	63.2		76.5(68.8)	
	2013	9846	21.7	66.6		74.1(65.1)	
	2014	10,795	23.8	67.7		68.5(64.7)	
Peripherality Index of locality	Highly peripheral	3660	10.2	61.0	0.001>	78.2(75.1)	0.001>
	Medium	9259	25.8	65.4		71.8(69.0)	
	Centralised	23,036	64.1	62.9		78.4(67.8)	

^a^SES −1-5 lowest, 16–20 highest

^b^District – Managerial district in the HCO

As shown in Table 1, adherence to colonoscopy was positively associated with socio-economic status (p0.001>), and inversely associated with age (p0.001>). Adherence was significantly lower in the Arab population (p0.001>). Participants living in highly peripheral or highly centralised localities were less likely to adhere than those living in localities in the medium peripherality index. (p0.001>). Participants living in the Southern District were more likely to have follow-up than those living in other districts.

The mean time to colonoscopy among participants was 76.2 days (SD 67.2) and the median time was 55 days. The number of days to colonoscopy was higher among Arab participants than Jewish ones (84 days vs. 77, *p* = 0.007) and higher in males than females (77.7 days, vs.75.9, *p* = 0.026). Time to colonoscopy was also higher in the lower SES categories (lowest 83.5 days vs. 72.3 days for the highest, *p* = 0.000), older participants (80.9 days for the oldest group vs. 75.6 days for the youngest, *p =* 0.000), and those living in localities with a medium peripherality index (medium 71.8 days vs. 78.4 days for the highest, *p =* 0.000).

In terms of change over the study period, adherence to colonoscopy increased from 55.8 to 67.7% (*p =* 0.001) between 2010 and 2014, and mean time to colonoscopy decreased from 88.9 to 68.5 days (*p =* 0.000), over the same period.

In terms of access and availability to colonoscopy, we calculated two variables: the mean distance between the home address and the nearest gastroenterology clinic for those who completed colonoscopy and those who did not, and the mean waiting time for colonoscopy at the nearest clinic for both groups. No differences in mean distance could be found between for those who completed the procedure (5.06 kms; SD 7.00), and those who did not (4.81kms, SD 6.8) (two-tailed *p* = 0.092). No differences in mean waiting time for colonoscopy at the clinics closest to participants’ homes could be found in either of the two HCO’s (*p* = 0.058 and *p* = 0.693). The arithmetic mean was 71 days for those who had a colonoscopy and 70 for those who did not. In addition, 73% of participants who had a colonoscopy after a positive FIT had the procedure at a gastroenterology clinic that was not the clinic closest to their home address.

Using multivariate regression analysis, we examined adherence to colonoscopy, adjusted for age, SES, ethnicity, district and peripherality (Table 2). Arab populations were less likely to complete follow-up (OR 0.61, *p* < .001), compared to the Jewish secular sector, and the oldest age group (aged 71–75) was less likely to complete follow-up (OR 0.79, *p <* .001), compared to the youngest group (aged 50–55).

The highest SES group was more likely to complete follow-up (OR 1.45, *p<*.001), compared with the lowest group. No significant differences were found between other age groups or gender in the adjusted model. The middle peripherality level was more likely to complete follow-up compared to the Centralized locality level (OR 1.21, *p<*.001).

**Table 2:** Multivariate analysis of colonoscopy adherence at 1 year following a positive FIT

Variable	Values	OR	***p***-value
Gender	Females vs Males	1.04	.083
Age group	50–55	1.0	
	56–60	0.93	.061
	61–65	0.96	.199
	66–70	0.95	.182
	71–75	0.79	<.001
SES	1–5	1.0	
	6–10	1.18	<.001
	11–15	1.31	<.001
	16–20	1.45	<.001
Ethnicity	Jewish Secular	1.0	
	Jewish Orthodox	1.02	.631
	Arab	0.61	<.001
Districts	Central	1.0	
	Southern	0.96	.344
	Northern	1.32	<.001
	Jerusalem	0.98	.592
Peripherality Index	Centralized locality	1.0	
	Highly peripheral	1.01	.859
	Middle	1.21	<.001

### Telephone survey results

During 2015, 2521 members of both HCO’s met the inclusion criteria and had a positive FIT (6% of those aged 50–74 who performed the FIT). Of these, we randomly selected 1522 for this part of the study. The final sample included 773 participants (50.8%) who completed the telephone questionnaire, 346 (45%) of whom had a colonoscopy within 90 days of a positive FIT. There were no statistically significant differences between participants and those who refused to complete the telephone questionnaire in gender, age, ethnicity or SES.

Over half of participants (56.1%) were male, 34% were aged 50–60, 46.7% were aged 61–70, and 17.1% were over 70. Most participants were Jewish (89.8%) and 72.7% were married, and 42.1% were born in Israel. One-fifth (20.4%) had less than 12 years education, 55.6% had completed 12–15 years education, and 24% 16 years and over. One third of participants reported being in excellent or very good health, and 7.9% in very bad health.

As can be seen in Table 3, no significant differences were found between those who had a colonoscopy and those who did not in age, gender, religiosity, education, marital status, country of birth, health status or SES. Patients who completed colonoscopy follow-up were significantly more likely to have complementary insurance (88.8% vs. 83.4%, *p* = 0.034) and private health insurance (49.1% vs. 35.9%, *p* < 0.001).

**Table 3:** Socio-demographic variable distribution by adherence to colonoscopy at 90 days

Variable	Value	Did not have colonoscopy ***N*** = 427	***N*** = 346	***p***-value
**Gender**	Male	57.1	54.8	0.506
	Female	42.9	45.2	
**Age**	50–55	15.4	18.1	0.373
	56–60	17.8	16.9	
	61–65	19.6	22.8	
	66–70	25.3	25.9	
	71–75	19.1	14.7	
**Religiosity**	Secular	54.2	54.4	0.797
	Traditional	31.0	30.9	
	Religious	9.6	8.1	
	Orthodox	5.2	6.7	
**Country of birth**	Israel	40.7	43.8	0.392
	Other	59.3	56.2	
**Ethnicity**	Jewish	88.1	91.7	0.108
	Arab	11.9	8.3	
**Education**	0–11	22.5	17.8	0.198
	12–15	53.0	58.8	
	16+	24.5	23.4	
**Marital status**	Single	6.5	4.4	0.500
	Married	71.9	73.7	
	Divorced	14.3	13.0	
	Widowed	7.3	8.9	
**PAM Level**	1	12.9	11.7	0.564
	2	13.2	10.4	
	3	22.0	21.1	
	4	51.9	56.9	
**Private insurance**	Yes	35.9	49.1	0.001
	No	64.1	50.9	
**Complementary insurance**	Yes	83.4	88.8	0.034
	No	16.6	11.2	
**Health status**	Excellent	10.1	6.9	0.369
	Very good	21.6	23.5	
	Good	34.0	32.7	
	Average	25.6	30.1	
	Bad	8.7	6.9	

We found no significant associations between patient activation level (PAM) and adherence to colonoscopy, using univariate and multivariate regressions.

**Fig. 1:**
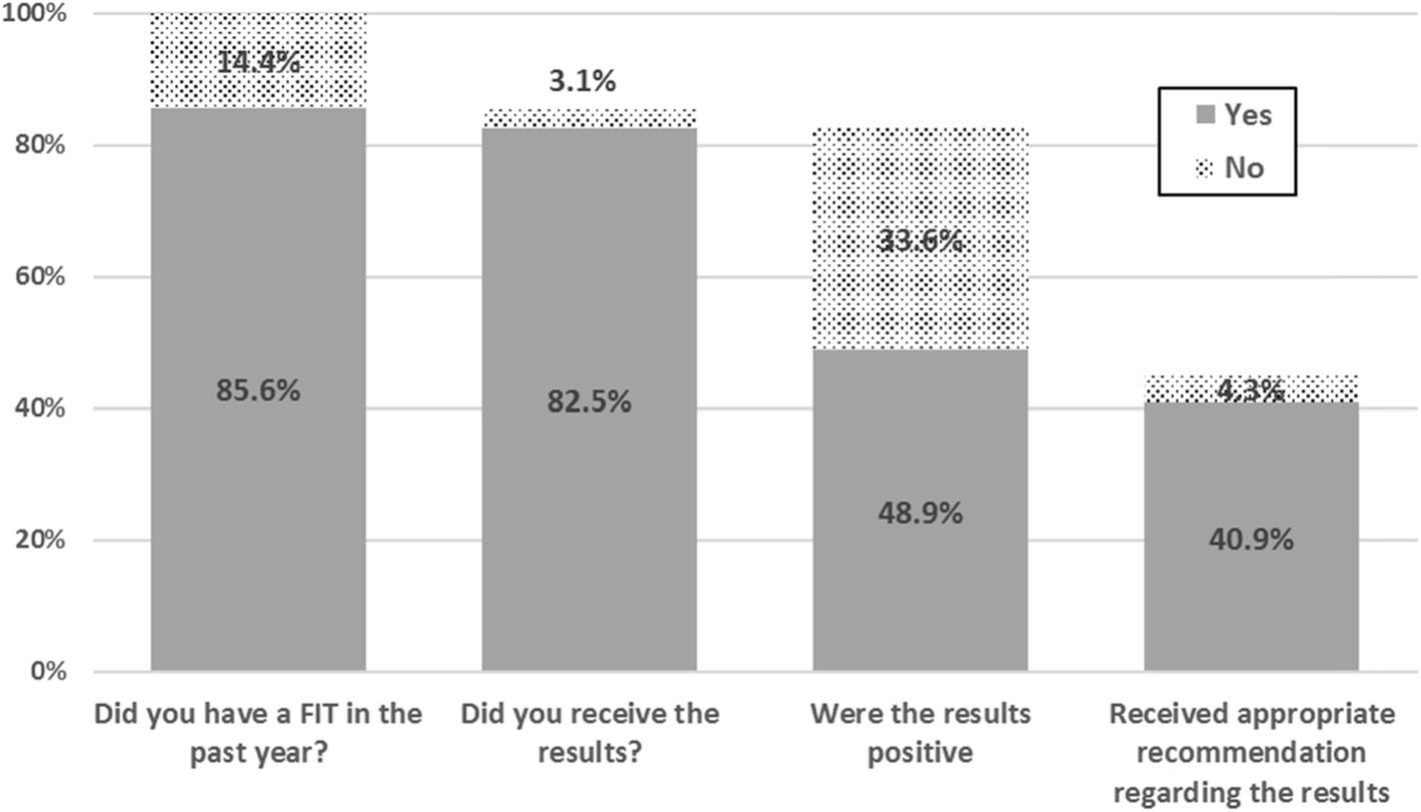
Proportions of participants who reported receiving information about the FIT*. *Each column represents the proportion of those who answered “Yes” in the previous column. Of those who recalled having a FIT, the proportion who recalled receiving the results, of those who recalled receiving the results, the proportion recalled the results being positive, and of those who recalled positive results, those who recalled receiving follow-up instructions

Participants were also asked whether they had completed a FIT during the past year, had received the results, whether the test was positive, and whether they had received any recommendations regarding test results. Less than half (40.9%) of the participants reported having completed a FIT, receiving a positive result and receiving appropriate recommendations (Fig. 1). In addition, 95.4% of responders stated that they had completed follow-up, and among those who did not complete follow-up the proportion was 87.4% (*p* = 0.001).

Participants who had a colonoscopy up to 90 days after a positive FIT were significantly more likely to remember having the test (88.4% vs. 83.4%, *p* = 0.028), receiving the results (98.7% vs. 94.38%, *p* = 0.002), being advised of the positive result (65.6% vs. 51.8%, *p =* 0.002), and receiving appropriate advice regarding follow-up (96.3% vs. 83.7%, *p* = 0.000).

We conducted multivariable regression analyses of the odds of having a colonoscopy within 90 days. After adjusting for age, gender, education, ethnicity and complementary insurance, participants with low comprehension were significantly less likely to have follow-up (OR = 0.52, 95%CI 0.37–0.71, *p* = 0.000), compared with participants with high comprehension.

Regarding the process of providing the test kit, there was no correlation between the profession of the provider of the FIT (General Practitioner (GP), gastroenterologist, nurse or lab technician) and follow-up. Most participants (82%) reported receiving instructions on the kit, and there was no difference between those who completed follow-up and those who did not.

We also asked about participants’ access to a gastroenterology clinic for the procedure. Over half (56%) responded that they had scheduled an appointment within 1 month or less. Two-thirds (66%) stated that clinic choice was based on the gastroenterologist performing the colonoscopy rather than distance, and 42.3% had made this selection based on their primary physician recommendation.

### Focus groups and in-depth interviews

There were five main themes in the information obtained from primary physician focus groups:

- **Lack of Awareness:** primary physicians perceived the problem to be much smaller than it is in reality, and felt that most of their patients with a positive FIT had completed follow-up. “This is very surprising! I was sure that it was a small number of patients … “ “I have been living under the illusion that all my patients complete follow-up”.
- **Workload:** primary physicians feel overworked, and this is one of the main reasons that they do not attend sufficiently to continuity of care: “I am very busy, every few minutes I have a patient with new problems, and I can’t always control what is happening. There are periods when I don’t have time for anything”.
- **Organizational prioritization:** follow-up after a positive FIT is one of many issues they deal with. It is not perceived as having major importance, and not a topic that they seek updated scientific knowledge on. They also do not feel that the organization places a great deal of importance on this. Physicians stated that there is not enough technological support such as alerts and reminders, and that patient telephone numbers are unavailable or incorrect at times. They also stated that long waiting times are an important barrier to colonoscopy.
- **Responsibility:** in terms of responsibility, physicians discussed two issues- the balance between patient and physician, and the balance between primary physicians and gastroenterologists. The primary physicians expressed frustration regarding patient autonomy: “ … it is 100% the responsibility of the doctor and the patient … on the one hand there is patient autonomy, very strong autonomy over their body, it is 100% theirs. On the other hand, the norms we live with in the modern world, the courts say that the doctor and the organization have a responsibility, we have to follow up and convince [the patient], and that is why it is 100% for both. In the end, it doesn’t matter who is responsible, if your patient is missed, you won’t sleep for a few nights.” Physicians also stated that once the FIT is positive, it is more the responsibility of the gastroenterologists to follow-up on the results.

#### Patient prioritization

physicians feel helpless when a patient refuses to have a colonoscopy. They find it challenging to be proactive, and do not have the skills necessary to engage the patients in preventive health care when the patient is seeking curative care. “Everything is important, but not necessarily to the patient … after the patient has stated the reason for his visit if you don’t fulfill their needs they may not return”. “There is nothing that is compulsory and that the patient **must do!** It is a matter of give and take … “ “Some patients will leave you if you offer them too many tests, and some want too many, which is very common”. In addition, the purpose of the consultation has nothing to do with CRC screening, and physicians find it challenging to introduce the subject.

Gastroenterologists did not express the same degree of surprise by the lack of follow-up by patients. They believe that most of the patients who did not have follow-up were erroneously given a FIT due to a “bug in the system”, and had had a colonoscopy prior to the positive FIT. In addition to this, they believe that fecal occult blood tests are unreliable and all relevant patients should have a screening colonoscopy “the occult blood test is unnecessary … if everyone had a colonoscopy it would save the organization money”.

Eight opinion leaders in the health system were interviewed. In general, two main themes arose: interviewees did not feel that the issue of follow-up after a FIT is very important, and they stated that the solution is to add this to existing quality indicators.

An interesting issue that arose was the friction between the public health/primary care approach and the gastroenterology professional association approach: “colonoscopy vs. fecal occult blood is a controversy between professional associations. The public health world says that the right [screening] test should be fecal occult blood, and people should have a colonoscopy when all else fails. The gastroenterology people have a different view and both sides will kill over their views. In practice, this caused paralysis for years with the effort to promote occult blood tests, while explaining that [screening] colonoscopy is bad and has lots of complications. Things are quieter now, they found a way to live together, they discredit each other less, and they accept the other side with a degree of equanimity.” Another interviewee stated: “the occult blood test is the primary screening [tool] because it is effective and good as a screening test … .it is not meant for 100% accuracy. It provides good primary mapping. There were arguments with the gastroenterologists and today they understand that it is essential because the alternative is expensive, complicated, deters from patient quality of life for three days and can lead to terrible overload in the system.”

## Discussion

Although there has been increased interest in follow-up colonoscopy after a positive fecal occult blood test in the past 2 years [41–44], this study is the first to investigate the barriers to colonoscopy following a positive FIT from a broad perspective, using both quantitative and qualitative methods, in a large population from two health care organizations. The aim of this study was to identify organizational, clinical, geographic, demographic and motivational barriers to undergoing a colonoscopy following a positive fecal occult blood test in a large population.

### Patient-related barriers

One of the most important findings of this study is that a large proportion of patients do not fully comprehend the reason for the FIT, and the implications of positive results. Approximately 40% of participants in our survey did not fully recall having the FIT, receiving the results, or understanding what needs to be done to complete the process with a positive FIT result, in spite of having documentation in their EMR attesting to having the FIT and being informed of the results. Both in univariate and multivariate analyses, this was a significant predictor of non-adherence to follow-up. These findings are in line with previous studies of screening for CRC, where lack of knowledge [10] and lack of understanding of the screening process [14] were strongly associated with non-adherence, and replicated findings from a previous study conducted in Meuhedet [45].

Emotional barriers to follow-up colonoscopy have been found in previous studies. Fear of the result may cause stress that prevents action in patients [29, 30]. A Canadian study described higher levels of anxiety related to the invasiveness of the procedure, and embarrassment among women, whereas men were more likely to fear the perceived loss of control associated with the procedure [31]. In a Japanese study, lack of knowledge, and stress related to the test results were the main barrier to screening [14]. Lack of trust in the patient-physician relationship has also been identified as a barrier [32]. In addition, misconception regarding the risk is also a factor, with many patients not being aware of the consequences of test results or not being appropriately informed by their physicians [10, 33]. Fear of the procedure was an important factor, as demonstrated in a study where those who refused follow-up colonoscopies had follow-up when offered a virtual colonoscopy [34].

It is possible that our finding that patients did not remember having the test or receiving the results reflects emotional issues that led to denial, rather than lack of comprehension, or a mixture of both.

We also found that lower socio-economic status, being a member of the Arab population, and in the oldest age group (71–75) were all associated with lower adherence to follow-up, and a longer period to colonoscopy among those who did have one. Previous studies have also found a significant association between older age and non-adherence to follow-up [41, 43], but not with ethnicity [41]. In Japan patients were more likely to be screened if they were older and male [14]. In an Israeli study of follow-up after a positive FOBT, younger patients were less likely to complete follow-up, as were those with chronic health conditions [35]. No clear association has been demonstrated between CRC screening follow up and SES, but one recent study by Martin et al. found that the level of medical insurance was significantly associated with follow-up, even though the study was conducted in a population where the follow-up is provided free of charge [43].

An interesting finding is that patient motivation, as measured by the PAM, was not associated with follow-up, as reported in a previous study [45]. This finding differs from a recent study of FOBT adherence in Iran [45] where self-efficacy was found be a strong predictor of screening behavior. Although PAM was not specifically measured, self-efficacy is associated with motivation, as is measured in the PAM.

Patient awareness and comprehension are essential to adherence to care recommendations. These are both associated with patient literacy [10, 33, 46], which, in turn is associated with education, SES, ethnicity and age. It may be that understanding the test and the results are a critical step for adherence, and therefore override patient motivation. Ensuring patient comprehension of the test, results and implications are the responsibility of the health care provider, and it appears there is need for improvement in this area.

### Provider related barriers

Most of the literature on the issue of completion of follow-up in screening programs has focused on physician and patient variables. One recurrent issue is lack of referral: physicians who don’t refer patients with positive FOBT to colonoscopy. Some of the reasons for this were associated with clinical decisions - patients who had a colonoscopy prior to the FOBT or patients who had other significant morbidities [25, 26]. Other studies found that lack of time and/or awareness of guidelines were associated with low follow-up rates [27, 28]. Patients of physicians who were more experienced, worked in group practices and in urban/central areas were more likely to have higher follow-up rates [23].

In our study, both primary care physicians and gastroenterologists underestimated the proportion of patients with a positive FIT who do not complete follow-up, and in particular PCP’s, who believed all their patients completed follow-up. As CRC screening is a national quality indicator reported annually by HCO’s [46] the focus of activity is completion of the FIT, rather than follow-up. In both HCO’s in this study, FIT kits are provided to patients by a variety of healthcare professionals, including nurses and laboratory technicians. We found no association between follow-up and the professional who provided the kit. There appears to be a certain degree of disengagement between the PCP’s and the screening process, as they do not feel responsible for the results, although they are both clinically and legally responsible. In contrast, the gastroenterologists stated they see the PCP as responsible for follow-up. In addition, they do not perceive fecal occult blood testing to be reliable, and would prefer all patients to have a colonoscopy.

PCP’s also stated that they feel that the importance of colorectal screening is not stressed enough in their daily routine – this may also be related to the provision of kits to the patients by other healthcare professionals.

### System related barriers

Access to colonoscopy and waiting times were not associated with follow-up. We found no difference in distance from the patient’s home to the closest gastroenterology clinic, or waiting times in these clinics, and follow-up. In previous studies access was found to be significantly associated with follow-up rates [23, 24]. This does not appear to be a significant problem in Israel, in spite of increasing quantities of screening tests.

Physician workload and lack of support arose as a major issue in the qualitative part of our study. This finding is similar to that of a study looking at follow-up barriers among physicians in the US within the Veterans’ Affairs system, where technical problems in the infrastructure between the laboratory, the physician and the healthcare provider resulted in delays in follow-up and diagnosis [22].

An important system barrier raised by physicians and opinion leaders is the lack of clarity and consensus regarding the FIT and its effectiveness. It is very possible that this ambiguity is conveyed to the patients, and contributes to their lack of comprehension discussed previously.

## Recommendations

1. It is essential to ensure that patients who are provided with a FIT understand the purpose of the test and what they need to do if the result is positive in advance. Reminders after completion of the test have differing degrees of effectiveness, although we found, in a previous study that telephone text reminders can increase follow-up in our population [9]. If patients are aware of the implications of non-adherence, reminders may prove to be more effective.
2. It is essential that the physician will make a reminder and talk with the patient about the importance of performing the test. In this way, the physician will also be able to discuss barriers and if necessary they will be able to help overcome those barriers.
3. Populations at risk for non-adherence should be identified, such as low SES and groups with cultural barriers. Tailored, culturally appropriate intervention need to be implemented in the populations to reduce health disparities.
4. Health care organizations need to have a program infrastructure in place to follow and support the screening process from start to end, using methods such as automated reminders for both physicians and patients, patient navigators, direct access to colonoscopy and priority appointments for patients with positive FIT [42].
5. Increase system, professional and public awareness of the importance of continuity of care for CRC screening.

### Study limitations

This mixed methods study has several limitations. The first is that it was conducted in two HCO’s with different policies and practices. In the analyses we treated all participants as one population. Another possible limitation is that in the retrospective population analyses we used a cutoff period of 1 year for follow-up, whereas in the case-control survey we used 90 days. In our initial analysis of all patients in the retrospective study we were unaware of the impact of waiting times, and therefore selected a generous window of opportunity for follow-up- 1 year. Having identified a median of 55 days to colonoscopy and a mean of 70.1 days, we based the second part of the study- our survey- on MOH guidelines – up to 90 days following a positive FIT. It is possible that this affected our findings in some way.

Another limitation of this study was that SES was not measured directly – it was obtained from the SES of the participants’ home address area based on postal codes. It is possible that an individual with high income or education levels resides in a low SES address. However, this methodology is common in research conducted as part of the Community Quality Indicator program in the Israeli Ministry of Health [8]. Ethnicity data was based on the ethnic affiliation of the clinics that patients attended rather than individual data, as these are not available to us. It is therefore possible that there is some misclassification in this variable, particularly for those living in large cities with mixed ethnic populations.

Waiting times for colonoscopy were obtained by contacting each gastroenterology clinic by telephone. It is possible that waiting times changed over the year of the study (2014) and the year we collected the information (2017), but the information was used for both participants with and without follow-up and therefore did not create a bias.

## Conclusion

This is one of the broadest study to date on the barriers to follow-up after a positive FIT. We identified important and innovative barriers at the patient, provider and system levels that need to be addressed through a systematic screening program with targeted interventions for populations at risk for non-adherence.

## Data Availability

Data for this manuscript will be available from the corresponding author upon reasonable request.

## Abbreviations

CI: Confidence Interval
CRC: Colorectal Cancer
EMR: Electronic Medical Record
FIT: Fecal Immunochemical Test
FOBT: Fecal Occult Blood Test
GIS: Geographic Information System
GP: General Physician
HCO: Heath Care Organization
IRB: *Institutional Review Board*
*IT*: *Information Technology*
OR: Odds Ratio
PAM: Patient Activation Measurement
PCP: Primary Care Physician
SD: Standard Deviation
SES: Socioeconomic status
